# The Meaning of Pain

**Published:** 1892-08-06

**Authors:** 


### The Meaning of Pain.

"Human reason, in one sphere of its cognition, is called upon to consider questions which it cannot decline, as they are presented by its own nature, but which it cannot answer, as they transcend every faculty of the mind." This dictum of Kant's rises to one's mind at the first glimpse of any treatise that deals with the subject of pain. So much has been written and said by teachers, preachers, philosophers, and poets, about pain, that one cannot but marvel how little of it all has been to the purpose. But they have all dealt more or less exclusively with the mystery of pain, accepting it as a mystery and leaving it one. The nearest approach to an explanation has been the statement that pain is the punishment, the revenge inflicted by outraged nature for any neglect or defiance of her laws. This is a truth, but an incomplete one; pain is the result of an unnatural condition of body, but neither God nor nature, His minister, descend to the malice of futile and meaningless revenge.

What then is pain? This is the question which Dr. Cameron Gillies* has set himself to answer in the able and philosophical pamphlet before us. Pain is not primarily a punishment, though when it comes, as a consequence of self-indulgence and sin, it may be so regarded. But in exactly the same fashion does it come to those who wrong nature against their will, to those who suffer from hunger and cold, to those who, for the sake of daily bread, must work long hours in poisonous atmospheres. The shop girl, the factory hand, the coal miner, are "punished" by disease as truly as the drunkard and the sensualist. We must go deeper than this for the meaning of pain. Much more satisfactory is Dr. Gillies' explanation that pain is a protest and a monitor; a protest against acts and conditions which are physiologically wrong; a monitor pointing out the necessity for change in the conditions that produced it. The demand becomes more and more peremptory, up to a certain point. But when pain ceases without any cessation of the conditions that produced it, it does not necessarily follow that the system has ceased to suffer, that any true condition of tolerance has been attained. Nature has given up her unheeded protests because they are no longer availing. "Ephraim is joined to his idols; let him alone," is the hardest of all sentences, for the letting alone means death.

This, then, is one use of pain—the beacon use, to show where danger lies. But though valuable enough in this alone to justify its existence, pain has a more distinct and affirmative value. We cry out when we are hurt because there is some sudden interruption of the machinery of the body. The word machinery is not in all respects a happy one, for machinery has no consciousness, and Descartes, carrying the notion to an extreme, declared that the lower animals suffered no conscious pain, and that their cries when being killed were only a mechanical noise caused by the breaking of the machine of the body—a notion which should be pleasing to vivisectors. But machinery implies that harmonious working together of varied parts which constitutes what we call life. When this is interrupted in any degree there is, to begin with, a sharp, half-mechanical cry—a cry of pain, indeed, but that pain due largely to surprise and bewilderment; a declaration by the brain that it has not yet realised the new condition of affairs. Then comes a pause. If the injury is severe we are stunned, insensible for a while, till the nerves have, so to speak, grasped the situation. Then, having apprehended this, they set to the work of repair, and pain begins again. This pain is a demand for material to build up the injured structure, and the request is granted in the form of inflammation—an apparent excess of tissue building matter. The inflammation relieves the pain by supplying the want it expressed; as it goes on the pain diminishes and disappears, and only when the wound is thoroughly mended does the inflammation disappear.

The connection between pain and strength was observed to be of such regular occurrence that ignorant people, keen-eyed as to results but blind to causes, inferred that pain was in itself directly helpful, and invented the proverb, "Pain is a tonic." Some people, who should not have been ignorant, skilful surgeons indeed, objected to anæsthetics because of some such notion. Dr. B. W. Richardson relates how some operators declared that the intense pain of a surgical operation "kept the patient up," and feared that when this fierce stimulant was removed by ether or chloroform he might die of collapse. The notion appears merely stupid and barbarous now, but it was simply a wrong inference from obvious facts. Some patients suffered intensely and recovered; others suffered less and died. They gave the credit to the pain itself, not to the more powerful vitality that was expressed by the keener suffering.

Pain, then, is life—life under wrong conditions and protesting against them, but emphatically the opposite of "the peace of death." It is the commonest of truisms that convalescents are infinitely more troublesome than those who are seriously ill. Their restlessness and irritability—real suffering of a kind—are proof of returning health. In fatal diseases we may see the opposite of this. In cases where the patient has suffered prolonged and acute pain, there comes at last an absolute cessation. He thinks, perhaps his family think, that the crisis is over and that he will recover. The crisis is indeed over, for nature has given up fighting a losing battle, and is now resting while the exhausted machinery runs out. We cannot tell what keen agony may be compressed in a moment in those deaths which we term instantaneous; but in serious accidents the pain is often in no way proportionate to the magnitude of the injury, and in fatal accidents there is often little or no pain.

In this connection, Dr. Gillies' remarks on what is called "Shock" are interesting, original, and suggestive. He calls it "Nature's time of grace." The word shock is applied to the insensibility following on an accident. If it is fatal in its consequences the patient may never recover consciousness, and people say, especially if no great outside injury be visible, that "he died of the shock." It would be more accurate to say he died in shock—in that natural anæsthesia produced by severe injury to the vital parts, from which nature, knowing that the end was death, did not think it necessary to awake him. Where recovery is possible, shock s temporary, and it is a common practice to shorten it by forcing stimulants down the patient's throat. "To restore him to consciousness" is the one aim of those around, even though consciousness means pain. Dr. Gillies suggests, and his theory commends itself to common sense, that this period of shock or insensibility is the time when remedial measures—the setting of a limb, the sewing of a wound—should be taken; it is nature's anæsthesia, which if destroyed may have to be reproduced by chloroform or something of the kind.

While Dr. Gillies believes that pain is valuable as an indicator and guide, he is by no means inclined to accept it as a "necessary evil," still less as a thing which is, save by way of protest, of divine origin. Therefore he protests against the so-called "natural pains" of child-birth and teething. Healthy women, healthy children, suffer little in these processes, he says, ; therefore, he argues, the ideally healthy mother or child would suffer not at all. But there is no record of a time even in the most arcadian age when these pains were not. "In sorrow shalt thou bring forth," if it be not a divine ordinance, expresses a conviction founded, even in the days of Moses, on universal and traditional experience. We can hardly believe that even in this the millennium will come. But that does not hinder us from appreciating this serious attempt of a clear and original mind to explain and profit by the lessons of pain.

* The Interpretation of Disease. Part I. "The Meaning of Pain," by H. Cameron Gillies, M.B. (London, David Nutt, 270-271, Strand. Price one shilling, nett).

